# Discrepancy between Lung Function Measurements at Home and in the Hospital in Children with Asthma and CF

**DOI:** 10.3390/jcm9061617

**Published:** 2020-05-26

**Authors:** Frederick L.G.R. Gerzon, Quirijn Jöbsis, Michiel A.G.E. Bannier, Bjorn Winkens, Edward Dompeling

**Affiliations:** 1 Department of Paediatric Pulmonology, School for Public Health and Primary Care (CAPHRI), Maastricht University Medical Centre (MUMC+), 6202 AZ Maastricht, The Netherlands; rickgerzon@hotmail.com (F.L.G.R.G.); r.jobsis@mumc.nl (Q.J.); michiel.bannier@mumc.nl (M.A.G.E.B.); 2 Department of Methodology and Statistics, CAPHRI, MUMC+, 6229 HA Maastricht, The Netherlands; bjorn.winkens@maastrichtuniversity.nl

**Keywords:** home monitoring, lung function, asthma, cystic fibrosis, children, telemedicine

## Abstract

The Coronavirus pandemic stresses the importance of eHealth techniques to monitor patients at home. Home monitoring of lung function in asthma and cystic fibrosis (CF) may help to detect deterioration of lung function at an early stage, but the reliability is unclear. We investigated whether lung function measurements at home were comparable to measurements during clinical visits. We analysed prospectively collected data of two one-year observational cohort studies in 117 children (36 with CF and 81 with asthma). All patients performed forced expiratory volume in one second (FEV_1_) measurements with a monitor at home. Paired FEV_1_ measurements were included if the measurement on the home monitor was performed on the same day as the FEV_1_ measurement on the pneumotachometer during a two monthly clinical visit. Bland-Altman plots and linear mixed model analysis were used. The mean difference (home measurement was subtracted from clinical measurement) in FEV_1_ was 0.18 L in CF (95% confidence interval (CI) 0.08–0.27 L; *p* < 0.001) and 0.12 L in asthma (95%CI 0.05–0.19 L; *p* < 0.001). FEV_1_ measurements at home were significantly lower than clinically obtained FEV_1_ measurements, which has implications for the application of this technique in the daily clinical situation.

## 1. Introduction

Pulmonary function tests play an important role in the monitoring of asthma and cystic fibrosis (CF) in children [1,2,3]. In asthma, the main treatment goals are good symptom control and an optimal lung function, in combination with a low risk on exacerbations [4]. Insufficient treatment may lead to airway remodelling and decline in lung function. Therefore, international guidelines recommend lung function measurement at least annually, but more frequently in high-risk patients [4,5].

In CF, the natural history of lung disease is characterized by an increased decline in lung function with episodes of acute worsening during pulmonary exacerbations [6]. Besides clinical assessment, pulmonary function tests are important to monitor lung disease progression at least four times a year [7].

In general, pulmonary function tests in patients with asthma and CF are performed in the hospital, but as telemedicine is finding its way into mainstream medical practice, home monitoring of lung function could be a helpful additional tool to detect early changes in lung function [8]. This may be particularly helpful in the early detection of exacerbations and may also decrease the frequency of hospital visits. Moreover, the current COVID-19 pandemic increased the need for eHealth techniques to monitor chronic patients at home, in order to prevent hospital visits and increased risks on contamination and infection.

Various studies showed that a home spirometer can provide reproducible and technically acceptable measurements [9,10,11]. However, these studies compared a home spirometer with a hospital spirometer (mostly pneumotachograph) in the clinical setting, there are no studies directly comparing lung function measurements at home to lung function measurements in the hospital. Other studies have evaluated home spirometry in combination with electronic symptom assessment as a tool to assess pulmonary exacerbations in children with CF, and to measure asthma control in children with asthma [12,13,14]. The accuracy of lung function measurements at home in these studies was unclear.

For clinical practice it is particularly useful to know the agreement between these two types of lung function measurement.

Therefore, the aim of our study was to investigate whether the forced expiratory volume in one second (FEV_1_) measurements at home were comparable to clinical FEV_1_ measurements. We hypothesised that lung function measurement using a home spirometer in the home setting will provide significant lower FEV_1_ values than clinically obtained lung function measurements using a pneumotachometer. This hypothesis is based on the knowledge that lung function tests are highly effort dependent and there are no trained medical personnel in the home setting who can encourage patients to perform lung function measurement with maximum effort.

## 2. Materials and Methods

### 2.1. Study Design

We performed a re-analysis of prospectively collected data of children with asthma or CF who were included in two previous one-year observational cohort studies [12,13]. In the ‘Reduction of Asthma exacerbation rate in Children by Non-invasive Monitoring of Inflammatory Markers in Exhaled Breath (Condensate)’ study (RASTER) (clinicaltrials.gov, NCT 01239238), the aim was to investigate the predictive properties of markers in exhaled breath to predict an asthma exacerbation. In addition, the reliability of home monitor assessments, including symptoms and lung function, to measure asthma control was investigated [12].

In the ‘Prevention of CF Exacerbation in Childhood: Early Recognition of Inflammation by Non-invasive Biomarkers in Exhaled Breath (Condensate)’ study (PREVEC) (clinicaltrials.gov, NCT 01241890) the aim was to assess the predictive properties of inflammatory markers in exhaled breath for pulmonary exacerbations in children with CF. In addition, the reliability of home monitor assessments of symptoms and lung function was investigated [13].

In the studies used for this study, ethics approvals were obtained from the Medical Research Ethics Committee of the Maastricht University Medical Centre. The studies were conducted in accordance with the relevant guidelines and regulations. Written consent was obtained from the parent or legal guardian and from children aged 12 years and older [12,13].

### 2.2. Patients

Extensive and specific details about the diagnostic in- and exclusion criteria of children with asthma and CF were previously published by van Vliet et al. [12] and van Horck et al. [13].

In short: children in the RASTER cohort fulfilled the diagnostic asthma criteria (including asthma symptoms in combination with reversibility to a beta-2 agonist) according to the Global Initiative for Asthma (GINA) and the Dutch Paediatric Pulmonology Society (SKL) [15]. In the PREVEC cohort, CF was defined as the presence of characteristic clinical features (persistent pulmonary symptoms, meconium ileus, failure to thrive, steatorrhea) in combination with an abnormal sweat test (chloride ≥ 60 mmol/L) and/or two CF mutations.

For the present analysis, available lung function measurements were only included if the FEV_1_ measurement at home was performed on the same day as the FEV_1_ measurement during a clinical visit, in order to prevent disease variation from influencing the outcome. Baseline characteristics of the patients in this study are shown in Table 1. The baseline characteristics are based on hospital pulmonary function tests.

### 2.3. Lung Function Measurements

In both studies all patients performed FEV_1_ measurements at home with a home spirometer. Patients were asked to use the home spirometer at least three times per week at the same time of the day. Patients had regular clinical doctor visits every two months where clinical lung function measurements were performed [12,13]. For our study we used the FEV_1_ measurements starting two months after the first clinical visit because instructions on home spirometer use were not given before the first visit.

### 2.4. Hospital Measurements

During the clinical visits dynamic spirometry was performed by the Masterscreen^TM^ Pulmonary Function Testing unit (Pneumotachograph, Vyaire Medical, Houten, The Netherlands) according to American Thoracic Society (ATS) and European Respiratory Society (ERS) standards [12,13,16]. Daily calibration of the instrument was performed at the lung function laboratory. The highest value of three technically correct maximal expiratory flow volume curves was used for analysis. Patients were instructed to stop use of short-acting bronchodilators at least 8 h and long-acting bronchodilators at least 48 h before testing.

### 2.5. Home Monitor

Lung function tests at home were performed using an electronic handheld spirometer, the AM2+ (CareFusion, Houten, The Netherlands). Lung function measurements consisted of three forced vital capacity (FVC) manoeuvres (with maximum effort). From this measurement the maximum FEV_1_ was used for analysis. Patients were instructed to always use the home monitor on the same time of the day.

In accordance with the ATS/ERS guidelines we defined an acceptable difference between tests when the difference between the largest FEV_1_ in both tests was equal or less than 0.150 L [17].

### 2.6. Statistical Analysis

Numerical variables are expressed as mean with standard deviation (SD) or as median with inter quartile range (IQR). Categorical variables are expressed as number and percentage.

In order to compare both devices on an individual level, Bland-Altman plots were made with the difference in FEV_1_ values between both measurement methods on the *y*-axis and the mean FEV_1_ value of the two methods on the *x*-axis. Linear mixed model analysis was performed to estimate the overall mean difference in FEV_1_ values between the two measurement methods, where a random intercept on child level was included to account for the correlation between repeated measures within the same child. To assess the change in difference scores over time (possible learning effect), the visit number was added as a fixed factor to the model. To account for individual differences in slopes (visit effect), a random slope was also considered, where the random intercept (RI) and random slope (RS) where allowed to be correlated. The best structure (RI+RS correlated, RI+RS uncorrelated, RI only) was based on the Bayes information criterion (BIC). A similar linear mixed model as for learning curve was done for age (numerical; in years) to see whether the difference scores were dependent on age. Linearity assumption was checked by testing for a quadratic (centered) age effect. If this linearity assumption was violated, not only the linear, but also quadratic trend was reported. Data were analysed with IBM SPSS Statistics for Windows version 25.0 (Armonk, NY, United States: IBM Corp.).

## 3. Results

### 3.1. Patient Characteristics

One hundred and seventeen patients were included in this study: 81 with asthma and 36 with CF (Table 1). 13 children with CF of the PREVEC study by van Horck et al. [13] and 15 of the children with asthma of the RASTER study by van Vliet et al. [12] were excluded from analysis because they had no simultaneous FEV_1_ measurement with the home spirometer and the hospital pneumotachograph on the same day. In total, 86 paired lung function measurements at various time points were selected from the CF group, and 321 paired lung function measurements at various time points in children with asthma were selected.

### 3.2. Comparison of Home Monitor FEV_1_ Measurement with FEV_1_ Measurement Using Pneumotachometer in Hospital Setting in Patients with CF

**Figure 1:**
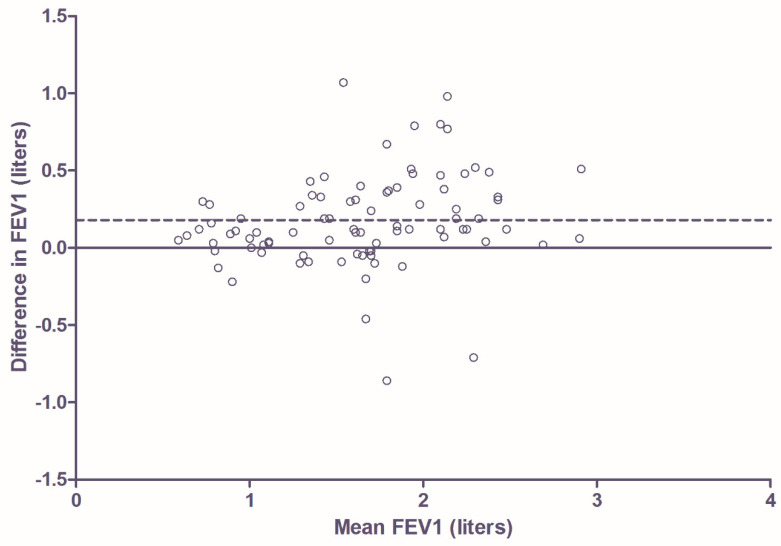
Bland-Altman plot showing degree of agreement between the two types of measurement in patients with CF. Difference in FEV1 is calculated by subtracting home FEV1 measurement from clinical FEV1 measurement. The dotted line shows the mean difference (0.18 L).

In children with CF, the FEV_1_ was significantly lower in the home measurements compared to hospital measurements (mean difference 0.18 L; 95% CI 0.08–0.27 L; *p* < 0.001). The Bland-Altman plot (Figure 1) showed the degree of agreement between the two methods. Difference in FEV_1_ was calculated by subtracting the home FEV_1_ measurement from the clinical FEV_1_ measurement. In this figure a total of 86 paired measurements are shown. As described in Section 2, these are not all of different participants because multiple paired measurements of an individual participant could be collected at various time points. In the 36 children with CF, 25 children (69%) had multiple measurements at different time points. Seventy of the 86 paired measurements (81%) had a difference of 0.05 L or more. Fifty-seven (66%) had a difference of 0.10 L or more and 43 of the 86 measurements (50%) had a difference of 0.15 L or more. There was no significant learning effect (overall *p* = 0.406), but the difference in FEV_1_ increased significantly with age (mean increase = 0.04 L/year, 95% CI 0.01–0.07, *p* = 0.011).

### 3.3. Comparison of Home Monitor FEV_1_ Measurement with FEV_1_ Measurement Using Pneumotachometer in Hospital Setting in Patients with Asthma

**Figure 2:**
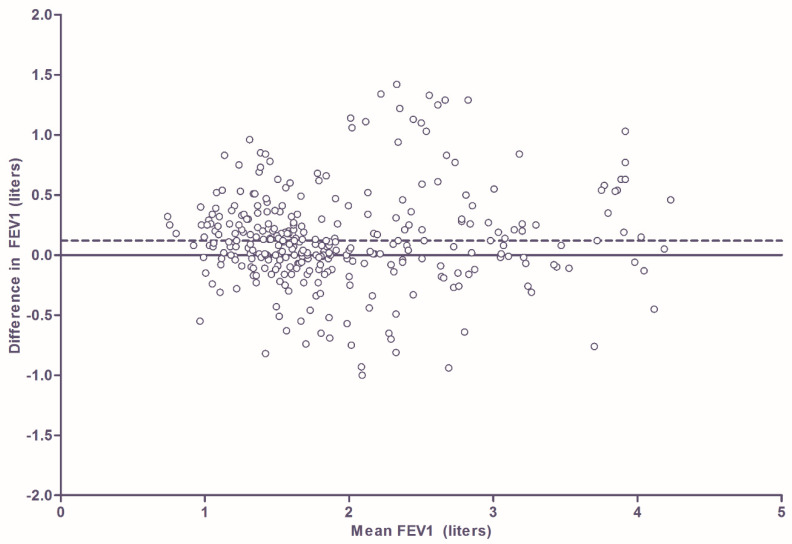
Bland-Altman plot showing degree of agreement between the two types of measurement in patients with asthma. Difference in FEV1 is calculated by subtracting home FEV1 measurement from clinical FEV1 measurement. The dotted line shows the mean difference (0.12 L).

With asthma, the FEV_1_ was significantly lower in the home measurements compared to hospital measurements (mean difference 0.12 L; 95% CI for difference 0.05–0.19 L; *p* < 0.001). Bland-Altman plot (Figure 2) shows the degree of agreement between the two devices. In this figure a total of 321 paired measurements are shown. As was the case in the CF population, not all measurements are of different participants because multiple paired measurements of an individual participant could be collected at various time points. In the 81 children with asthma, 70 children (86%) had multiple paired measurements at different time points. Two hundred and sixty-five of the 321 paired measurements (83%) had a difference of 0.05 L or more. Two hundred and twenty-six (70%) had a difference of 0.10 L or more and 186 (58%) of the 321 measurements had a difference of 0.15 L or more. There was no significant learning effect (overall *p* = 0.281), but the difference in FEV_1_ increased significantly with age (mean increase = 0.03 L/year, 95% CI 0.01–0.05, *p* = 0.003).

## 4. Discussion

To our knowledge, this is the first study comparing FEV_1_ measurements using a portable spirometer in the home setting to FEV_1_ measurements by a pneumotachometer in the hospital. We performed a re-analysis of two prospective long-term studies in paediatric asthma and CF and assessed the degree of accordance between home monitoring versus clinical monitoring of lung function. Our study showed that home FEV_1_ measurements with a portable spirometer in children with asthma and CF were significantly lower than assessments with a pneumotachometer in the hospital at the same day. The degree of difference between home and hospital FEV_1_ was comparable for asthma and CF. A bigger difference in FEV_1_ than the predetermined acceptable difference of 150 mL was present in 58% of the patients with asthma and in 50% of the patients with CF.

The current COVID-19 pandemic increased the need for new eHealth techniques to monitor patients at home.

Such an approach may contribute to social isolation measures and may prevent hospital visits with an increased risk of COVID-19 contamination, subsequent infection, and further dissemination of COVID-19. In patients with chronic lung diseases like asthma, cystic fibrosis and chronic obstructive pulmonary disease (COPD), there is a lot of interest in home monitoring of symptoms and lung function.

It has been shown that it is possible to record spirometric parameters in school children unsupervised at home following comprehensive training [18]. To accomplish this, participants in our study had clinical visits every two months, during which the technique of FVC manoeuvres with maximum effort on the home spirometer was evaluated and instructions for use at home were given. Even though this critical evaluation was repeated every two months, the significant difference between the two methods of FEV_1_ measurements remained in the course of the studies. There was no influence of multiple measurements in the course of time, which indicated absence of a learning effect.

Our findings are to some extent in accordance with previous studies, which also suggested a significant lower FEV_1_ with a home spirometer compared to the hospital FEV_1_ on the pneumotachograph [9,10]. However, our study gives a better reflection of the ‘real life’, daily clinical practice: in earlier studies, the home monitor was tested in a clinical setting, where trained laboratory personnel was present so that best performance on the home monitor could be established.

In our ‘real life’ study, the home monitor was used in the home setting, where no medically trained personnel were present to encourage patients to perform the lung function measurements with maximum effort and to determine whether measurements were adequate. Therefore, our study gives a better reflection of the reliability of home monitoring of lung function in daily practice. The systematic lower values of FEV_1_ as assessed by the electronic home monitor compared to the clinically assessed FEV_1_ could not be explained by a low accuracy of the first instrument. In a previous study, the measurement of PEF, FEV1 and FVC by means of the home monitor satisfied the criteria for monitoring devices of the ATS, including the criteria for inter- and intradevice reproducibility [19]. Although this study also reported lower FEV1 values of the home monitor compared to a pneumotachograph, our results indicate a bigger difference between these two devices in a ‘real life’ clinical setting.

Our data showed an effect of age. The mean difference in FEV1 in both the CF and asthma cohort increased significantly with age. There can be several explanations for this. One is that parents had a positive effect on the quality of lung function measurement at home by means of encouragement and that the influence of parental encouragement was greater in the younger age group than in older children. Another possible explanation is that younger children have a smaller FEV1 and FVC, and therefore the mean difference in FEV1 could also be less than in older children who in general have bigger FEV1 and FVC values. 

Our study had some limitations. Although all patients were asked to always perform lung function measurements at home on the same time of the day, we did not have access to the specific time of the day that the measurements were actually performed. Therefore, we do not know how much time passed between the two measurements on the same day, which may have slightly contributed to the differences between the home and the hospital assessments because of the diurnal variability of lung function parameters. It is also possible that some patients performed the FEV1 measurement on the home spirometer after the measurement with the pneumotachometer in the hospital, although this was not the study routine. This is relevant because in the hospital FEV1 measurement was performed before and after short acting bronchodilators. In our study we used the FEV1 measurement of the pneumotachometer before use of bronchodilators for comparison of the devices. In theory it is possible that patients performed the home spirometer measurement after they received bronchodilators in the hospital. This could imply that the significant difference that was already found could in fact be an underestimation.

Do our findings imply that there is no role for home monitoring of lung function in children with asthma or CF? In our view, there certainly is a role for home monitoring of lung function when used to determine lung function trends in the assessment of pulmonary exacerbations in patients with CF or asthma control in patients with asthma, as was shown in the previous studies by van Horck and van Vliet [12,13]. These studies respectively showed that the combination of home monitor FEV_1_ measurements and Respiratory Symptom Scores (RSS) in the four weeks to one week before a Pulmonary Exacerbation (PEx) could discriminate between children who remained stable and children who developed a PEx, and that by means of the home monitor, twice as many cases of partly controlled asthma were found than with the hospital Asthma Control Questionnaire (ACQ).

However, our data suggest that, at least in children, home monitoring of lung function is still inferior to clinical measurement of lung function. Therefore, home monitoring should not replace lung function testing during clinical visits at this moment.

To improve the possibility of more accurate lung function measurements at home, various supportive actions could be considered. For example, FVC manoeuvres could be performed under the direct supervision of trained personnel at home or under supervision at a distance (for example with a video connection with the clinical centre). Another option is using more technically advanced portable spirometers which can provide direct feedback on the performed FVC manoeuvres, therefore potentially increasing the accuracy of the performed manoeuvres. There already is a variety of these kind of portable spirometers commercially available as was shown in the recent review by Carpenter et al. [20]. However, more studies should be conducted on the usability and accuracy of these devices.

## 5. Conclusions

Our study showed that in children with asthma and CF, home FEV_1_ measurements with a portable home spirometer were significantly lower than FEV_1_ measurement by a pneumotachometer in the hospital. Therefore, the use of a home spirometer in the follow up of children with asthma or CF should be critically evaluated. Further (prospective) studies are needed in which the use of lung function measurements at home are directly compared to clinical lung function measurements, and the effects of various methods to improve the reliability are compared.

## Figures and Tables

**Table 1:** Patient characteristics.

	Baseline Characteristics	
	CF (*n* = 36)	Asthma (*n* = 81)
Age (y), mean (SD)	9.4 (2.8)	9.6 (3.0)
Male sex, *n* (%)	22 (61)	43 (53)
FEV_1_ (L), mean (SD)	1.70 (0.53)	1.91 (0.76)
FEV_1_%pred, mean (SD)	87.3 (17.0)	97.0 (14.3)
FVC%pred, mean (SD)	91.9 (16.3)	98.5 (11.6)
FEV_1_/FVC, mean (SD)	0.81 (0.09)	0.84 (0.08)
Homozygous dF508, *n* (%)	25 (69)	n.a.
Pseudomonas colonisation, *n* (%)	12 (33)	n.a.
BMI, median (IQR)	16.6 (16.0–17.6)	n.a.
Prophylactic antibiotics, *n* (%)	22 (61)	n.a.
ICS use, *n* (%)	11 (31)	75 (93)
ICS + LABA use, *n* (%)	n.a.	33 (41)
Atopic, *n* (%)	n.a.	62 (76)

CF: cystic fibrosis; FEV_1_: forced expiratory volume in 1 s; FVC: forced vital capacity; ICS: inhaled corticosteroids; BMI: body mass index; LABA: long acting beta agonists; n.a.: not available.
